# *Staphylococcus aureus* Protein A Plays a Critical Role in Mediating Bone Destruction and Bone Loss in Osteomyelitis

**DOI:** 10.1371/journal.pone.0040586

**Published:** 2012-07-11

**Authors:** Amro Widaa, Tania Claro, Timothy J. Foster, Fergal J. O’Brien, Steven W. Kerrigan

**Affiliations:** 1 Microbial Infection Group, Molecular and Cellular Therapeutics, Royal College of Surgeons in Ireland, Dublin, Ireland; 2 Department of Anatomy, Royal College of Surgeons in Ireland, Dublin, Ireland; 3 Department of Microbiology, Moyne Institute of Preventive Medicine, Trinity College Dublin, Dublin, Ireland; 4 Trinity Centre for Bioengineering, Trinity College Dublin, Dublin, Ireland; 5 School of Pharmacy, Royal College of Surgeons in Ireland, Dublin, Ireland; Harvard Medical School, United States of America

## Abstract

*Staphylococcus aureus* is the most frequent causative organism of osteomyelitis. It is characterised by widespread bone loss and bone destruction. Previously we demonstrated that *S. aureus* protein A (SpA) is capable of binding to tumour necrosis factor receptor-1 expressed on pre-osteoblastic cells, which results in signal generation that leads to cell apoptosis resulting in bone loss. In the current report we demonstrate that upon *S. aureus* binding to osteoblasts it also inhibits *de novo* bone formation by preventing expression of key markers of osteoblast growth and division such as alkaline phosphatase, collagen type I, osteocalcin, osteopontin and osteocalcin. In addition, *S. aureus* induces secretion of soluble RANKL from osteoblasts which in turn recruits and activates the bone resorbing cells, osteoclasts. A strain of *S. aureus* defective in SpA failed to affect osteoblast growth or proliferation and most importantly failed to recruit or activate osteoclasts. These results suggest that *S. aureus* SpA binding to osteoblasts provides multiple coordinated signals that accounts for bone loss and bone destruction seen in osteomyelitis cases. A better understanding of the mechanisms through which *S. aureus* leads to bone infection may improve treatment or lead to the development of better therapeutic agents to treat this notoriously difficult disease.

## Introduction

Bone tissue is composed of both mineral and organic material designed specifically for strength and rigidity to support the load-bearing structure of the body. Bone is constantly undergoing remodelling from birth to death. This is a complex process involving bone formation followed by bone resorption. The bone remodelling process is tightly controlled by the coupled action of osteoblasts and osteoclasts that sequentially carry out formation of new bone followed by resorption of old bone [1].

Bone formation results from a complex cascade of events that involve proliferation of primitive mesenchymal stem cells, differentiation into matrix forming osteoblasts and finally mineralisation. During the osteoblast maturation phase, several markers of osteoblast growth and division (osteoblastogenesis) are expressed including alkaline phosphatase and type I collagen, both of which are important for bone matrix deposition and mineralisation [2], [3]. When fully differentiated, mature osteoblasts also produce regulators of matrix mineralisation such as osteocalcin, osteopontin and osteonectin [4]. Bone resorption is mediated by activated multinucleated osteoclasts [5] that are derived from mononuclear precursor cells of the monocyte-macrophage lineage in the bone marrow [6]. Receptor activator of nuclear factor (NF)-kB ligand (RANKL) is the dominating cytokine regulating osteoclast differentiation and proliferation (osteoclastogenesis). It is produced predominantly by osteoblasts in membrane-bound and soluble forms [7].

Bone is a sterile organ system that is highly resistant to bacterial infection. However, a small number of organisms have a predilection for the skeleton. Such breaches can lead to serious bone disease such as septic arthritis [8] and osteomyelitis [9] which often cause serious morbidity [10]. Bacteria can reach the bone by haematogenous spread, direct inoculation or from a contiguous focus of infection. The bloodstream may be invaded from a breach in the skin, infected wound or infected umbilical cord. Direct inoculation of bone can occur from penetrating injuries, open fractures, joint replacements and surgical contamination. Contiguous sources may occur when infection is transmitted from local tissue in the cases where infection in diabetics spread from soft tissues to bone [11]. Inflammation, which often accompanies infection, compresses the vasculature thus preventing immune cells from reaching the infected area. Bone devoid of blood supply detaches from the healthy bone to form a sequestrum which is inaccessible to immune cells [12]. Treatment is often unsuccessful as the infected nidus that harbours sessile matrix-protected pathogens is impermeable to antibiotics and often requires radial debridement coupled with systemic antibiotic treatment [13].

Although a broad range of bacterial species have been isolated in cases of septic arthritis, osteoitis and osteomeylitis, *Staphylococcus aureus* is the main offender, accounting for between 37% to 80% of cases [11]. *S. aureus* is a normal commensal of the human body and usually lives in harmony with its host without causing symptoms. Its primary habitat is the anterior nares in 20% of the population and is transiently associated with the rest [14]. The success of *S. aureus* as an opportunistic pathogen is due in part to its expression of a wide array of microbial surface components recognising adhesive matrix molecules (MSCRAMM’s) [15]. Using these MSCRAMM’s *S. aureus* can attach either directly or indirectly to host cells including bone cells [16], [17], [18], [19].

Protein A is an MSCRAMM and is an important virulence factor of *S. aureus* [14]. It binds a number of plasma proteins, including the Fc portion of IgG [20], the Fab-heavy chain of the Vh3 region of IgM molecules [21] and vonWillebrand factor [22], as well as tumour necrosis factor receptor-1 (TNFR-1) [23], [24] and the epidermal growth factor receptor (EGFR) [25]. SpA comprises an N-terminal ligand binding domain (E, D, A, B, C) linked to the cell wall peptidoglycan via the C-terminal LPXTG motif [26].

When bone tissue gets infected it upsets the normal process of bone remodelling that is orchestrated by osteoclasts and osteoblasts. While many attempts have been made to understand the mechanisms involved, most studies have focused on the effect *S. aureus* has on osteoblasts. Early studies demonstrated *S. aureus* fibronectin binding proteins (FnbpA and FnbpB) can bind to osteoblasts. This interaction leads to internalisation of *S. aureus* into phagocytic vesicles in the osteoblast [18]. This process renders the bacteria safe from either immune or antibiotic attack. More recently, our group demonstrated that *S. aureus* SpA can bind directly to pre-osteoblastic cells via Tumour Necrosis Factor Receptor-1 (TNFR-1) without the presence of an extra cellular matrix [19]. Engagement of this receptor results in signal generation that leads to osteoblast apoptosis preventing new bone formation [19]. While these studies provide critical information in the way osteoblast cells respond to *S. aureus* in bone formation, they did not explain effects on bone loss or resorption. In this paper we demonstrate that when *S. aureus* binds to osteoblast cells, in addition to inducing apoptosis, it stunts osteoblast cell growth (osteoblastogenesis). Furthermore, *S. aureus* also induces expression of soluble RANKL by osteoblasts which in turn recruits osteoclasts and promotes their formation and proliferation. This increases the bone resorptive power of osteoclasts which further weakens the skeleton. Deletion of SpA from *S. aureus* abolishes all of these responses suggesting that SpA is a double-edged sword, which inhibits osteoblast growth while at the same time increasing bone resorption. A better understanding of the mechanisms leading to bone infection may aid in the improved treatment or development of novel therapies to treat this disease.

## Results

### *S. aureus* Inhibits Osteoblast Proliferation Over a 21 Day Period

Previously we demonstrated that *S. aureus* SpA promotes adherence to pre-osteoblastic cells in non-osteogenic culture [19]. In order to further understand the nature of this interaction we investigated the ability of committed osteoblasts to proliferate in the presence of pro-osteogenic media conditions and *S. aureus* over a 21 day period. This was in order to demonstrate that *S. aureus* still inhibits proliferation in the presence of an extra cellular matrix produced by osteoblasts and that SpA/TNFR1 interaction occurs despite the presence of a matrix. *S. aureus* were formaldehyde fixed to prevent loss of essential nutrients necessary for osteoblast growth and proliferation. Consistent with previous results formaldehyde fixation did not have any effect on SpA as determined by Western immunoblotting [19]. SpA surface expression was confirmed on *S. aureus* strains Newman wildtype and the complemented strain, while Newman ΔSpA was deficient in this protein (data not shown).

**Figure 1:**
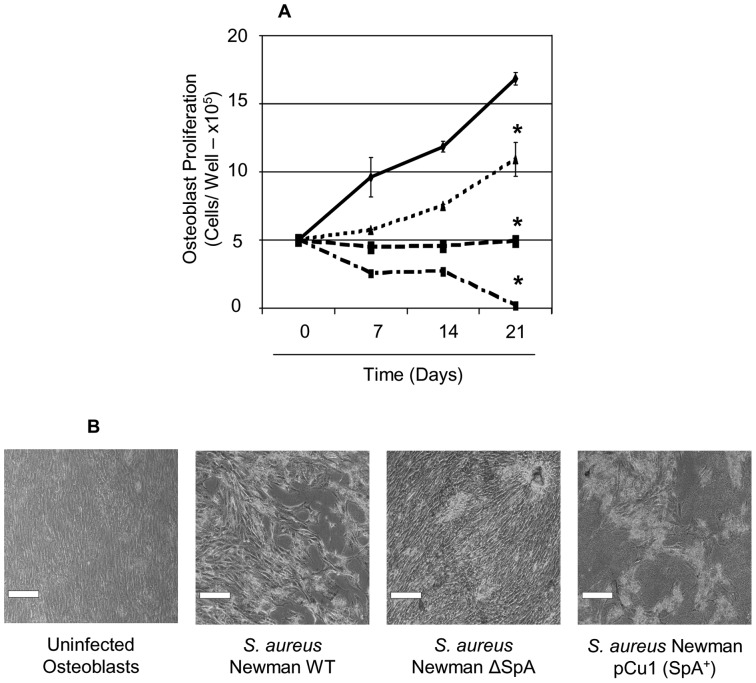
*Staphylococcus aureus* prevents osteoblast growth and proliferation over a 21 day period. Osteoblasts (5×10^5^ cells/ml) were preincubated with either control buffer (——) or formaldehyde fixed S. aureus Newman (-----), Newman Δspa (⋅⋅⋅⋅⋅), or Newman spa (pCU1spa^+^) (— ⋅ —). (**A**) Following days 7, 14 and 21 osteoblasts were removed by trypsinization and proliferation was determined by counting cells on a haemocytometer. (**B**) Osteoblasts were also visualised using an inverted bright field microscope (Leica, DMIL) at x200 magnification., *P<0.0001, n = 5.

Formaldehyde-fixed *S. aureus* were added to osteoblasts and cell counts were obtained at day 7, 14 and 21 using a haemocytometer (Figure 1A). Images were obtained using brightfield microscopy (Figure 1B). Uninfected osteoblasts proliferated normally over the 21 day period. Addition of *S. aureus* prevented osteoblasts from proliferation over the course of the 21 days compared to the uninfected osteoblasts (P<0.0001, n = 5). Addition of a mutant defective in expression of SpA allowed proliferation of osteoblasts significantly more than the *S. aureus* infected osteoblasts (P<0.0001, n = 5) but not as much as the uninfected osteoblasts (P<0.0001, n = 5). Complementation of *S. aureus* mutant with pCU1*spa* restored the inhibition of proliferation below levels observed in the wildtype (P<0.001, n = 5). These results collectively demonstrate that when *S. aureus* SpA binds to differentiating osteoblasts it prevents their growth and proliferation.

### *S. aureus* Inhibits Osteogenesis Markers Over a 21 Day Period

**Figure 2:**
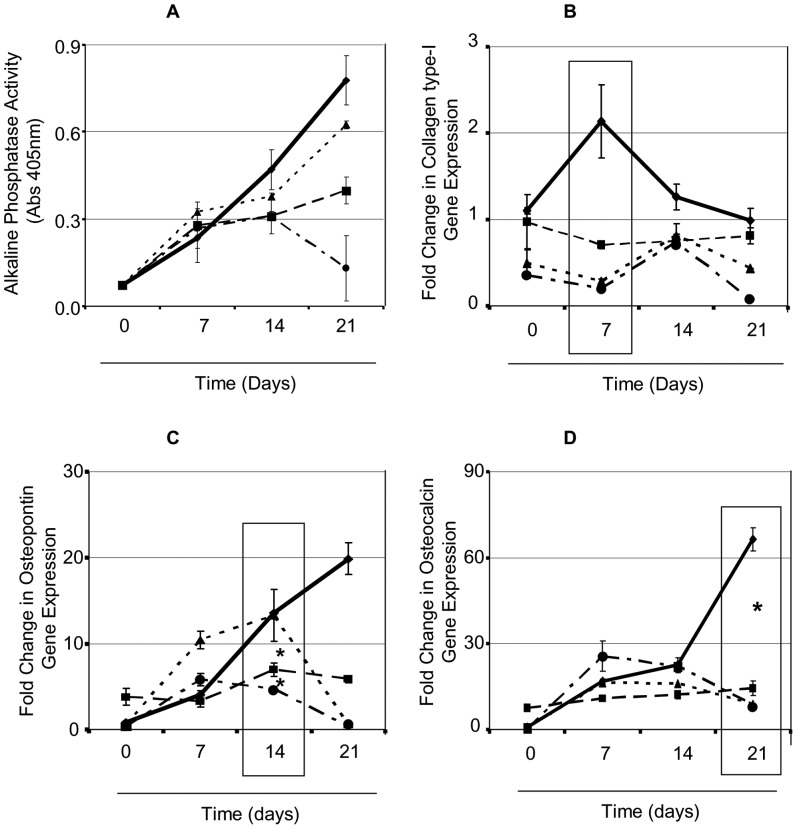
*Staphylococcus aureus* prevents expression of osteogenic markers. Osteoblasts (5×10^5^ cells/well) were incubated with either control buffer (——) or formaldehyde fixed *S. aureus* Newman (-----), Newman Δ*spa* (⋅⋅⋅⋅⋅), or Newman *spa* (pCU1*spa* ^+^) (— ⋅ —) for a period of 21 days. (**A**) Osteoblasts were lysed with 1 ml of lysis buffer containing a substrate for alkaline phosphatase (0.1 M Na acetate, 2% Triton X-100 and 10 mM p-nitrophenol phosphate) and incubated in the dark for 1 hour at 37°C. RNA was isolated and reverse transcribed to cDNA. Analysis of bone formation marker expression was carried out specifically for the early bone marker (**B**) collagen type I, (**C**) osteopontin and (**D**) osteocalcin. *0.01, **0.05, ***0.0001, n = 3.

We next investigated the effect *S. aureus* has on new bone formation (osteogenesis). Alkaline phosphatase is an enzyme found in osteoblasts and is a biochemical indicator of early stage osteogenesis. Osteoblasts were added to formaldehyde fixed *S. aureus* and the alkaline phosphatase content was measured at day 7, 14 and 21. Uninfected osteoblasts had a consistent increase in alkaline phosphatase over the 21 day period suggesting *in vitro* bone formation/mineralisation (Figure 2A). Addition of wildtype *S. aureus* Newman expressing SpA significantly inhibited alkaline phosphatase levels at day 14 and day 21 compared to uninfected osteoblasts (P<0.05, n = 3). In contrast to this, addition of a *S. aureus* mutant defective in expression of SpA failed to affect alkaline phosphatase at either day 7, 14 or 21 (P = NS, n = 3). The complemented mutant regained the inhibition effect to the same level as the wildtype *S. aureus* strain at day 7 and 14 compared to the uninfected osteoblasts (P<0.05, n = 3). At day 21 there was a significant drop in alkaline phosphatase content possibly due to osteoblast cell death. This may be explained by the over-expression of SpA on the surface of the complemented strain and the previous findings that *S. aureus* SpA can induce cell apoptosis.

During the process of osteogenesis, osteoblasts secrete proteins that form a matrix for newly proliferated osteoblasts to adhere to. Such matrix proteins include collagen type I, osteopontin and osteocalcin. We investigated the effect that *S. aureus* has on these osteogenic markers. To do this RNA was isolated from uninfected and *S. aureus-*infected osteoblasts following 0, 7, 14 and 21 days. Analysis of osteogenesis was carried out by quantitative real time PCR and the gene expression was calculated as a fold change compared with day 0 with either the uninfected or *S. aureus* infected osteoblasts.

Collagen type I is an early stage marker of osteogenesis that is typically expressed at day 7. Consistent with the literature there was a 2-fold change in expression of collagen type I in the uninfected osteoblasts at day 7 (Figure 2B). Expression gradually decreased back to baseline at day 14 and 21 in the uninfected osteoblasts. Addition of the wildtype *S. aureus* Newman strain, the *S. aureus* ΔSpA or the complemented mutant, to osteoblasts prevented the expression of collagen type I over 7, 14 or 21 days (P<0.0001; all strains versus uninfected, n = 3).

Osteopontin is a mid stage markers of osteogenesis. There was no significant increase in gene expression for osteopontin at day 0 for either the uninfected or *S. aureus-*infected osteoblasts (Fig 2C, P = NS, n = 3). However at day 14 there was almost a 10-fold increase in expression in uninfected compared to the *S. aureus-*infected osteoblasts (P<0.05, n = 3) and an almost 15-fold increase in expression in the uninfected osteoblasts at day 21 (P<0.01, n = 3). Interestingly addition of the *S. aureus* ΔSpA mutant induced higher expression of osteopontin at day 7 (P<0.01, n = 3) and a similar level of expression at day 14 compared to the uninfected osteoblasts (P = NS, n = 3). Expression of osteopontin was back to baseline levels on day 21 following addition of the mutant defective in SpA. Addition of the complemented strain failed to induce expression of osteopontin (compared to uninfected cells (P<0.0001, n = 3) with levels similar to the wildtype *S. aureus* Newman.

Osteocalcin is a late stage marker of osteogenesis. Expression was increased 18- and 25-fold respectively, at day 7 and 14 (Figure 2D, P<0.05, n = 3). At day 21 there was an almost 50-fold increase in osteocalcin expression in the uninfected osteoblasts. Addition of the wildtype *S. aureus* wildtype strain, the *S. aureus* ΔSpA mutant or the complemented mutant to osteoblasts prevented the expression of osteocalcin over 7, 14 or 21 days (P<0.0001; all strains versus uninfected, n = 3).

### *S. aureus* Induces RANKL Expression in Osteoblasts

**Figure 3:**
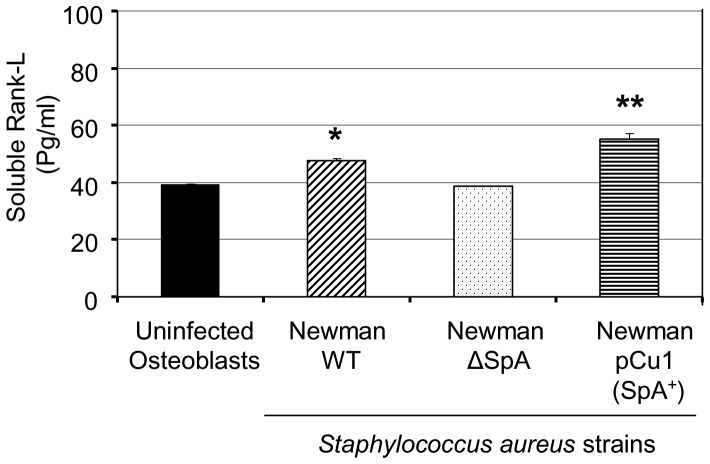
*Staphylococcus aureus* induces expression of soluble RANKL. Osteoblasts (1×10^6^ cells/well) were incubated with either control buffer, *S. aureus* Newman, Newman Δ*spa* or Newman *spa* (pCU1*spa* ^+^) for 24 hrs. Media from uninfected and infected osteoblasts was removed and centrifuged at x 10,000g for 2 minutes. Soluble RANKL was detected using an ELISA kit. *P<0.01, **P<0.001, n = 3

A common characteristic of infected bone tissue is widespread bone loss possibly due to increased bone resorption. RANKL is produced by osteoblasts and is the main trigger for recruitment of pre-osteoclasts and the subsequent trigger for bone resorption. We next investigated if RANKL is released from osteoblasts following infection with *S. aureus*. Quantitative analysis of soluble RANKL on uninfected osteoblasts demonstrated a low level of RANKL after 24 hrs. Addition of *S. aureus* Newman significantly increased RANKL expression after 24 hrs in the soluble fraction (Fig 3; P<0.01, n = 3). Addition of the *S. aureus* ΔSpA mutant failed to express significant levels of soluble RANKL compared to the uninfected sample (Fig 3; P = NS, n = 3). However, the complemented mutant led to a significant expression of soluble RANKL with levels significantly higher than the wildtype (Fig 3; P<0.001, n = 3).

### *S. aureus* Induces Migration of Pre-osteoclasts

**Figure 4:**
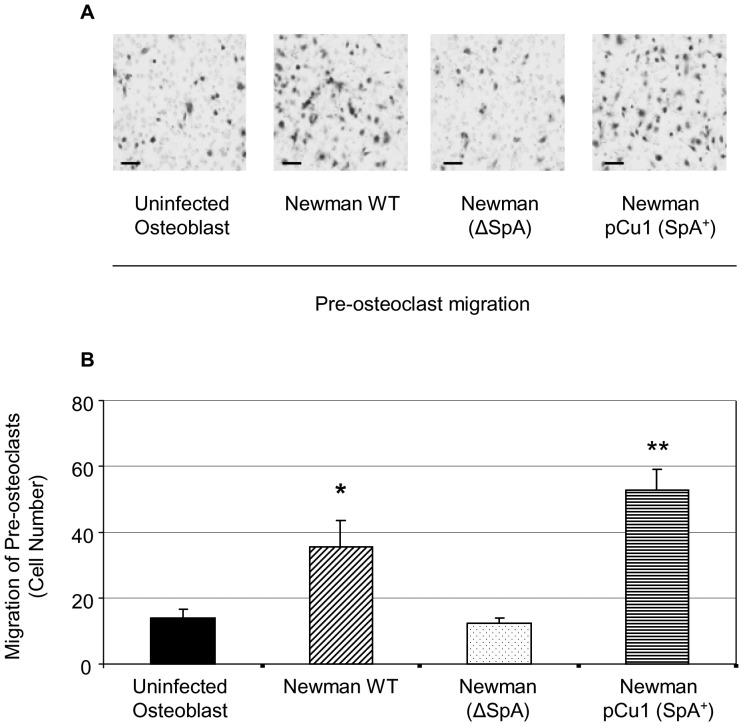
*Staphylococcus aureus* induces the migration of pre-osteoclasts. Osteoblasts were cultured for 4 days, in the presence and absence of *S. aureus*, before inserting hanging cell migration chamber inserts containing serum starved pre-osteoclasts. Following 18 hour incubation, membranes from the migration chamber were removed and fixed in 4% formaldehyde. The membrane was then stained with haematoxylin (**A**) and counted from 5 random fields of view (**B**). The average cell count was established as indicative of the total cell migration. *P<0.05, **P<0.001 n = 3.

We next investigated the migration of pre-osteoclasts toward *S. aureus*-infected RANKL-expressing osteoblasts. By adapting a previously described cell migration assay [27] it was possible to calculate the migration of individual pre-osteoclasts towards infected osteoblasts. Haemotoxylin stained pre-osteoclasts were counted from 5 random fields of view and the average cell count was established as indicative of the total cell migration. A small number of pre-osteoclasts migrated towards the uninfected osteoblasts following 18 hours of exposure (Figure 4A). Addition of *S. aureus* to the osteoblasts induced a significant increase in the number of pre-osteoclasts migrating in comparison to the uninfected sample (P<0.05, n = 3). Addition of the *S. aureus* ΔSpA mutant failed to induce migration of the pre-osteoclasts above uninfected levels (P = NS, n = 3). Finally addition of the *S. aureus* complemented mutant led to a significant increase in the migration of pre-osteoclasts (P<0.0001, n = 3).

### *S. aureus* Induces Osteoclastogenesis

**Figure 5:**
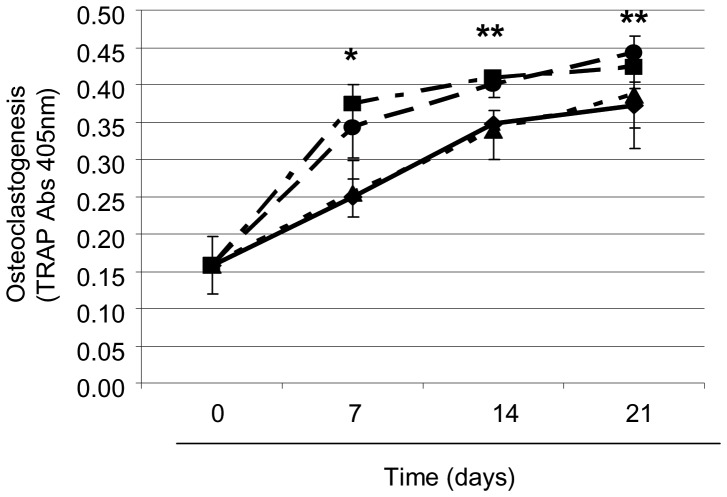
*Staphylococcus aureus* inhibits osteoclastogenesis. Pre-osteoclasts were seeded (2×10^4^ cells/well) in 12-well tissue culture plates and cultured for 21 days. Conditioned media from osteoblasts (4 days), with either control buffer (——) or formaldehyde fixed *S. aureus* Newman (-----), Newman Δ*spa* (⋅⋅⋅⋅⋅), or Newman *spa* (pCU1*spa* ^+^) (— ⋅ —) was then transferred to the pre-osteoclasts. Osteoclastogenesis was determined by measuring tartarate resistant alkaline phosphatase (TRAP). *P<0.05, **P<0.001 n = 5.

We next investigated whether *S. aureus* induced RANKL binding to the RANK triggers osteoclastogenesis. To do this the media was removed from osteoblasts infected with *S. aureus*, and transferred to the pre-osteoclasts for 7, 14 or 21 days. This conditioned media contains many secreted proteins from the osteoblasts, most notably, RANKL. Tartrate-resistant acid phosphatase (TRAP) is a glycosylated monomeric metalloenzyme that is highly expressed in osteoclasts and its detection is often used to measure osteoclastogenesis. As expected, the numbers of osteoclasts formed in the media from uninfected osteoblasts (Figure 5) rose steadily over the 21 day period. There was a significant increase in osteoclast formation following addition of *S. aureus* infected osteoblast media at day 7 (P<0.05, n = 5), 14 (P<0.0001, n = 5) and 21 (P<0.0001, n = 5). The *S. aureus* ΔSpA mutant reduced osteoclastogenesis to uninfected levels at day 7, 14 and 21 (P = NS, n = 5). Finally the numbers of osteoclasts growing in the media from the *S. aureus* complemented mutant were significantly increased and similar to the levels of the wildtype *S. aureus* Newman strain at days 7 (P<0.005, n = 5), 14 (P<0.0001, n = 5) and 21 (P<0.0001, n = 5).

## Discussion

Bone remodelling involves the coupled action of bone-forming osteoblasts and bone-resorbing osteoclasts. This process replaces old bone with new bone to ensure the integrity of the load bearing structure of the skeleton. This is a highly controlled sequence of events by both local and systemic factors, and any upset in the naturall balance between resorption and formation can lead to accelerated bone loss, resulting in an increased risk of fractures due to osteoporosis (which occurs due to an imbalance between formation and resorption) or another pathological disease. One potential mediator that can upset the process of bone remodelling is the presence of an infectious micro-organism. Although typically a sterile environment, trauma, bone surgery or placement of a foreign device such as an orthopaedic implant may expose this otherwise sterile environment to an infection. *S. aureus* is a common commensal of the human skin and mucous membranes and is the major pathogen isolated from patients with bone infection. The pathology of infected bone tissue shows severe inflammation, loss of vasculature and widespread bone destruction and bone loss. Recently we demonstrated that *S. aureus* SpA binds to the TNFR-1 which is expressed on osteoblasts [19]. This interaction results in a premature signal that triggers apoptosis. While this may account for some of the bone loss seen in infected bone tissue, it does not account for the failure of pre-osteoblasts to differentiate into fully mature matrix-producing osteoblasts or indeed for the widespread bone destruction seen in infected tissue. Therefore this study investigated additional factors that may contribute specifically to bone loss and bone destruction.

Bone formation or osteogenesis is typically characterised by the sequential expression of a series of bone formation markers including type I collagen, alkaline phosphatase, osteopontin and osteocalcin. Osteoblasts then eventually produce RANKL [28] which acts as a signal for the recruitment, proliferation and activation of osteoclasts which initiates the resorption phase. During staphylococcal bone infection a number of cytokines are released into the surrounding environment that critically affect bone formation [29], [30]. For example, TNFα is known to independently control apoptosis and regulation of cell proliferation, both of which contribute to loss of bone formation [31]. As TNFα is detectable only at low levels in osteoblasts [32] it is unlikely that it is responsible for the inhibition of osteogenesis that was observed in figure 2. Recently we demonstrated that *S. aureus* SpA interacts with osteoblastic cells directly, by binding to the TNF receptor [19]. This interaction triggers a proteolytic cascade, by recruiting and activating the initiator caspases 3 and 6, resulting in osteoblast apoptosis. The interaction between *S. aureus* SpA and TNFR-1 may help explain the current results. In the current study we found that *S. aureus* also inhibited osteogenesis by inhibiting osteoblast proliferation and expression of alkaline phosphatase, type I collagen, osteopontin and osteocalcin over a 21 day period under pro-osteogenic *in vitro* culture conditions. A *S. aureus* mutant deficient in SpA significantly recovered osteoblast alkaline phosphatase and proliferation, suggesting that *S. aureus* binding to the osteoblast TNF receptor controls these events similar to TNFα binding to its receptor. For example, Gilbert *et al*. demonstrated that TNFα binding to its receptor, TNFR1, inhibits osteoblast differentiation independently of apoptosis in mice [33]. The importance of this observation is critical to the understanding of the molecular mimicry that exists between bacterial cell wall surface proteins and known host factors.

Interestingly the cells infected with the *S. aureus* mutant deficient protein A failed to affect expression of type I collagen or osteocalcin. One reason for this might be the role IL-1β plays in bone formation. Several reports in the literature have demonstrated that IL-1β inhibits expression of osteocalcin and type I collagen [34], [35]. IL-1β can be induced in human and murine osteoblasts by a variety of stimuli including TNFα [36], [37], [38]. Indeed increased levels of IL-1β have been detected in several animal models of bone infection [39]. Collectively these results suggest that *S. aureus* SpA might be mimicking the effects of TNFα by binding to TNFR-1. This interaction maybe responsible for triggering apoptosis and inhibiting osteoblast proliferation and differentiation simultaneously. At the same time osteoblasts release the inflammatory cytokine IL-1β which inhibits expression of osteogenic markers critical for matrix deposition and mineralisation, thus collectively contributing to bone loss. Additional experiments are required to measure the release of IL1β following infection with *S. aureus* in order to confirm this link.

Widespread bone destruction is also characteristic of an infection in bone tissue and is typically mediated by activated multinucleated osteoclasts. During the bone formation process another cytokine, RANKL, is produced by osteoblasts which serves to modulate the activity or formation of osteoclasts [7]. RANKL is a 317 amino acid polypeptide that is expressed either on the surface of osteoblasts or released into the local environment, and binds to and activates the RANK receptor expressed on osteoclasts. Once activated, osteoclasts begin the resorption of bone. However, the process of bone resorption needs to be tightly controlled, as uncontrolled signals can lead to excessive bone destruction and cause severe weakening of the skeletal system. Consistent with previous results we found that *S. aureus* infection of osteoblasts led to an increase in RANKL expression in their membrane and also in a soluble form released into the surrounding environment [40]. Previously it has been demonstrated that expression of RANKL leads to the migration and formation of osteoclasts, which is suggestive that *S. aureus* induced bone infection initiates localised bone resorption. Here we demonstrate that osteoclasts migrate toward *S. aureus* infected osteoblasts releasing RANKL. More importantly, deletion of SpA from *S. aureus* completely abolished RANKL expression and ablated migration and proliferation of the osteoclasts. This is most likely because *S. aureus* SpA is unable to bind to osteoblast TNFR-1 to induce release or expression of RANKL. These results are consistent with the finding that TNFα binding to TNFR-1 on osteoblasts results in increased RANKL expression which triggers subsequent bone destruction [41], [42]. RANKL is most likely released with other pro-osteoclastogenic cytokines that induce bone resorption e.g. Interleukin 6 [29]. Whether *S. aureus* SpA regulates these other pro-osteoclastogenic or not requires further investigation.

A picture of the mechanisms that leads to bone infection is slowly starting to develop in the literature. Early reports demonstrated that as a defence mechanism, *S. aureus* can become internalised by osteoblasts. Uptake is promoted by fibronectin binding proteins that capture fibronectin and use it as a bridge between bacteria and the α5β1 integrin expressed on osteoblasts. Integrin clustering results in signalling that lead to bacterial uptake thus rendering the bacteria safe from both immune and antibiotic attack [18]. In the current study we used a strain of *S. aureus* (Newman) that does not express Fnbp’s on its surface [46]. In addition, our group previously demonstrated that *S. aureus* strain Newman can bind to osteoblasts even in the absence of fibronectin or FnbpA and FnbpB [19]. These results suggest that a second interaction between *S. aureus* and osteoblasts exists. Previously we demonstrated that an early step in *S. aureus* infection of the bone occurs when the major cell wall surface protein, SpA binds to TNFR1 on osteoblasts. This triggers a series of events that lead to induction of osteoblast apoptosis and inhibition of mineralisation [19]. These events may account for the bone loss seen at infected sites. In the current study we further develop these observations by proposing a model though which *S. aureus* infection of bone can also encourage bone resorption, thus accounting for bone destruction. It is essential that bone is constantly turned over in order to maintain strength and rigidity in the skeletal system. The process of bone remodelling is tightly controlled by a series of autocrine signals that control the correct amount of bone resorption versus bone formation. Any significant deviation in resorption and formation means accelerated bone loss and increased risk of fractures and breaks. In the present study we demonstrate that when *S. aureus* SpA binds to osteoblast TNFR-1 it prevents expression of osteogenic markers essential for matrix formation and bone formation (Table 1). Furthermore, binding of *S. aureus* SpA to TNFR1 induces the release of soluble RANKL, a cytokine critical for the migration of the bone resorbing cells, the osteoclast (Table 2). Deletion of SpA prevents these events from occurring. Put together these simultaneous signals will contribute to the extensive bone loss and bone destruction observed in tissue from bone infection patients. The final step in the infection process occurs when the fibronectin binding proteins facilitate the internalization of *S. aureus* into the osteoblast [18]. These internalized *S. aureus* have also the ability to induce apoptosis via the TRAIL/caspase pathway [43], [44], [45]. Given that a large percentage of clinical isolates obtained from osteomyelitis cases contain both protein A and the fibronectin binding proteins (both greater than 80%) collectively the current and previous mechanistic data suggest a multi-pronged approach is involved in bone destruction and bone loss. One limitation of this study is that all experiments have been carried out *in vitro* using cell culture techniques, further experiments using animal models are required to establish if these processes occur *in vivo*. A better understanding of the mechanisms that lead to bone infection *in vitro* are required before any *in vivo* experiments take place, such information may lead to improved therapies to treat this disease.

**Table 1:** Summary of the effects *Staphylococcus aureus* has on osteoblasts.

*S. aureus* strain	Proliferation	Alkaline Phosphatase	Collagen type I	Osteopontin	Osteocalcin
No bacteria (control)	+	+	+	+	+
Newman wildtype	–	–	–	–	–
Newman SpA-	+	+	–	+	–
Newman pCU1spa+	–	–	–	–	–

**Table 2:** Summary of the effects *Staphylococcus aureus* has on osteoclasts.

*S. aureus* strain	RANKL	OPG	Migration of Osteoclasts	Osteoclastogenesis
No bacteria (control)	–	+	–	–
Newman wildtype	+	–	+	+
Newman SpA–	–	+	–	–
Newman pCU1spa+	+	–	+	+

## Materials and Methods

### Bacterial Strains and Growth Conditions

*S. aureus* strains used in this study are listed in Table 3. Bacteria were grown overnight to the stationary growth phase at 37°C in Brain Heart Infusion broth (Sigma-Aldrich, Ireland). *S. aureus* strain Newman pCU1 (SpA^+^) was grown with the addition of 10 µg/ml chloramphenicol (Sigma-Aldrich, Ireland). *S. aureus* were fixed in 4.8% formaldehyde (Sigma-Aldrich, Ireland) under constant rotation for 10 minutes. Bacteria were then washed and isolated by centrifugation at 4,000 g for 5 minutes and re-suspended in phosphate buffered saline (PBS) (Sigma-Aldrich, Ireland) at pH 7.4. *S. aureus* were adjusted to 1×10^9^ cells/ml for all studies.

**Table 3:** List of strains of *Staphylococcus aureus* used in this study.

Strain or plasmid	Relevant Characteristics	Reference
***S. aureus***		
Newman	NCTC 8178 wildtype	Duthie and Lorenz, 1952
Newman *spa*	Newman *spa::*Ka^r^ defective in protein A	O’Brien et al, 2002
***Plasmids***		
pCU1*spa* ^+^	Shuttle vector capable of replicating in *E. coli* and *S. aureus*. Cm^r^ Ap^r^ *spa* gene cloned into pCU1	Claro et al, 2011

### Tissue Cell Culture Conditions

The mouse clonal MC3T3-E1 pre-osteoblastic cell line (ATCC, Middlesex, UK) was used for all experiments. This is a common cell line used routinely for investigating osteoblast function. The cells were cultured in standard T175 tissue culture flasks (Sarstedt, Ireland) containing α-MEM supplemented with 10% FBS (Biosera Ltd., United Kingdom), 2% penicillin-streptomycin solution and 1% L-glutamine (Sigma-Aldrich, Ireland). The media was replaced every 3–4 days and after confluency, cells were harvested using trypsin-EDTA (Sigma-Aldrich, Ireland) and re-suspended in the standard medium. MC3T3-E1 pre-osteoblastic cells were differentiated to matrix secreting mature osteoblasts using the standard osteogenic cocktail, by supplementation of dexamethasone (100 nM), ascorbic acid (50 µg/ml) and β-glycercolpentahydrate (10 mM) (Sigma-Aldrich, Ireland).

The murine RAW 264.7 pre-osteoclast cell line (ATCC, Middlesex, UK) was used for all osteoclast studies. This cell line is commonly used to assess osteoclast function. Murine RAW 264.7 cells were cultured in T175 tissue culture flasks (Sarstedt, Ireland) in high glucose Dulbecco’s Modified Eagles Medium (DMEM) (Sigma-Aldrich, Ireland) supplemented with 10% heat inactivated FBS (Biosera Ltd., United Kingdom) and 1% penicillin-streptomycin solution (Sigma-Aldrich, Ireland).

### Osteoblast Proliferation

*S. aureus* were allowed to adhere to 6 well plates for two hours at 37°C, in the presence of 5% CO_2_. To confirm uniformity of adhesion between the various *S. aureus* strains (Newman wildtype, Newman SpA-, Newman pCU1 SpA+) to the 6 well plates, bacteria were stained with crystal violet (5%) for 10 mins and read at 620 nm on a platelet reader. After 2 hours, unbound bacteria were removed by gentle aspiration. MC3T3-E1 cells (5×10^5^ cells/well) were then seeded onto the immobilised bacteria for 24 hrs. Following 24 hrs the standard media was replaced with osteogenic media. Osteoblasts were removed from the wells on days 7, 14, and 21 days by trypsinization. Following this the cells were pelleted by centrifugation and resuspended in 1 ml media. Osteoblast proliferation was determined by counting cells on a haemocytometer with a 1∶1 dilution with Trypan Blue (Sigma-Aldrich, Ireland), to distinguish between viable and non-viable osteoblasts.

### Assessment of Osteogenesis Markers

Alkaline phosphatase expression was evaluated by removing osteoblast media and washing in warm PBS. Osteoblasts were lysed with 1 ml of lysis buffer containing a substrate for acid phosphatase (0.1 M Na acetate, 2% Triton X-100 and 10 mM p-nitrophenol phosphate) and incubated in the dark for 1 hour at 37°C. The reaction was stopped using 0.3 M NaOH (Sigma-Aldrich, Ireland). Triplicates (100 µl) of each well were then added to a 96-well plate and absorbance was then read at A_405nm_, using a microplate reader (Wallac Victor2, United Kingdom).

RNA was isolated from osteoblasts on days 0, 7, 14 and 21 respectively. Isolation was carried out by lysing the cells in RLT lysis buffer (Qiagen, United Kingdom). The cell lysates were then centrifuged in QI Shredder columns (Qiagen, United Kingdom) and RNA was extracted using an RNeasy Mini Kit (Qiagen, United Kingdom) according to the manufacturer’s instructions. RNA concentration was quantified using a Nano-Drop (Mason Technology, United Kingdom) spectrophotometer (absorption 260 nm). Subsequent to RNA extraction and quantification, genomic DNA was removed and the RNA sample was reverse transcribed using 300 ng of total RNA with an RT kit (QuantiTect RT Kit, Qiagen, United Kingdom). Real-time polymerase chain reaction (PCR) was then conducted using a 7500 Real-time PCR System (Applied Biosystems, United Kingdom). The QuantiTect SYBR Green PCR Kit (Qiagen, United Kingdom) was used according to the manufacturer’s instructions, with QuantiTect Primers (Qiagen, United Kingdom). Analysis of bone formation marker expression was carried out specifically for the early bone marker collagen type I, the mid/late stage markers, osteopontin and osteocalcin (Qiagen, United Kingdom). Expression levels were assessed using the relative quantification DDCt method and all gene expression levels were normalised using the housekeeping control 18S ribosomal RNA (Qiagen, United Kingdom).

### Western Blot for Protein A

*S. aureus* Newman strains were lysed for 10 minutes on ice in RIPA buffer supplemented with 1x protease inhibitor cocktail (Sigma-Aldrich, Ireland). Protein lysate (10 µg) were separated on a 10% sodium dodecylsulfate polyacrylamide (SDS-PAGE) gel. Proteins were then transferred onto poly-vinyldifluoride membranes (Roche, United Kingdom) for 1 hour. The membranes were exposed to primary chicken anti-protein A IgY antibody (clone SPA-27) overnight under constant rotation at 4°C. Unbound antibody was removed from membranes by 10 minute washes (x3) with Tris buffered saline (TBS) - 0.1% Tween buffer. Protein bands were detected using species-specific horseradish peroxidase-conjugated secondary antibody and developed by chemiluminescence.

### Quantification of Soluble RANKL

MC3T3-E1 osteoblastic cells (1×10^6^ cells/well) were cultured for 7 days until confluency was reached and then cultured for a further 24 hours, in the presence and absence of *S. aureus*, before removing media which was placed on ice for analysis later. The resulting uninfected and *S. aureus* infected osteoblast media was centrifuged at 10,000 g for 2 minutes at 4°C to remove debris. Soluble RANKL was detected using an ELISA kit (R&D Systems, Minneapolis, MN) according to manufacturer’s instructions.

### Migration of Pre-osteoclasts

RAW 264.7 pre-osteoclastic cells (2×10^4^ cells) were seeded in 8 µm pore hanging cell culture inserts (Millipore, Ireland) and serum starved for 2 hours at 37°C, 5% CO_2_ in Optimem serum-free media (Invitrogen, Ireland). *S. aureus* (1×10^9^ cells/ml) were immobilised on 24-well plates and MC3T3-E1 osteoblastic cells (5×10^5^ cells/well) were seeded as previously described. Osteoblasts were cultured for 4 days, in the presence and absence of *S. aureus*, to allow sufficient release of chemotaxis factors, before inserting hanging cell migration chamber inserts containing serum starved pre-osteoclasts. Pre-osteoclast cells were then incubated for 18 hours at 37°C, 5% CO_2_ to assess migration through the porous membrane towards osteoblasts in presence and absence of *S. aureus*. Following 18 hour incubation, membranes from the migration chamber were removed and fixed in 4% formaldehyde. The membrane was then stained with haematoxylin for 10 minutes before mounting on a glass slide. Pre-osteoclasts were counted from 5 random fields of view and the average cell count was established as indicative of the total cell migration. All images were taken using an inverted bright field microscope (Leica, DMIL) at x200 magnification.

### Quantification of Tartate Resistant Acid Phosphate (TRAP) as a Measure of Osteoclastogenesis

To determine the proliferation of pre-osteoclasts, RAW 264.7 cells were seeded (2×10^4^ cells/well) in 12-well tissue culture plates in the presence of 100 ng/ml RANKL and cultured for 21 days. Conditioned 4 day media from osteoblasts, either exposed or unexposed to *S. aureus*, was then transferred to the pre-osteoclasts. TRAP was quantified to measure osteoclastogenesis at day 7, 14 and 21 respectively. TRAP quantification was carried out by removing conditioned media from pre-osteoclasts and washing cells in PBS. Cells were then incubated for 1 hour at 37°C in 1 mM citrate solution. After the hour incubation, 20 µl was added in triplicates to a 96-well plate containing 50 µl 100 mM p-nitrophenol phosphate, 80 mM sodium tartrate, 200 mM sodium citrate and 200 mM sodium chloride (Sigma-Aldrich, Ireland). After 30 minutes incubation at 37°C, the reaction was stopped using 1 M NaOH. Absorbance was then read at A_405nm_, using a microplate reader (Wallac Victor2, United Kingdom).

### Statistical Analysis

All statistical analysis was performed using a two-tailed Student’s t-test with the statistical software SSC-Stat V2.12. Data were represented as the mean values +/− the standard error of the mean (SEM). All values were considered significant at P<0.05.
